# Binaural beats at 0.25 Hz shorten the latency to slow-wave sleep during daytime naps

**DOI:** 10.1038/s41598-024-76059-9

**Published:** 2024-10-30

**Authors:** Zhiwei Fan, Yunyao Zhu, Chihiro Suzuki, Yoko Suzuki, Yumi Watanabe, Takahiro Watanabe, Takashi Abe

**Affiliations:** 1 International Institute for Integrative Sleep Medicine (WPI-IIIS), University of Tsukuba, 1-1-1 Tennodai, Tsukuba, Ibaraki 305–8575 Japan; 2 The Japan Society for the Promotion of Science (JSPS) Foreign Researcher, Tokyo, Japan; 3 Graduate School of Comprehensive Human Science, University of Tsukuba, Tsukuba, Japan; 4 KYOCERA Corporation, Kyoto, Japan

**Keywords:** Binaural beats, Slow oscillations, Slow-wave sleep, Slow-wave activity, Sleep stages, Neural entrainment, Human behaviour, Non-REM sleep, Sleep, Slow-wave sleep

## Abstract

Binaural beats can entrain neural oscillations and modulate behavioral states. However, the effect of binaural beats, particularly those with slow frequencies (< 1 Hz), on sleep remains poorly understood. We hypothesized that 0.25-Hz beats can entrain neural oscillations and enhance slow-wave sleep by shortening its latency or increasing its duration. To investigate this, we included 12 healthy participants (six women; mean age, 25.4 ± 2.6 years) who underwent four 90-min afternoon nap sessions, comprising a sham condition (without acoustic stimulation) and three binaural-beat conditions (0, 0.25, or 1 Hz) with a 250-Hz carrier tone. The acoustic stimuli, delivered through earphones, were sustained throughout the 90-min nap period. Both N2- and N3- latencies were shorter in the 0.25-Hz binaural beats condition than in the sham condition. We observed no significant results regarding neural entrainment at slow frequencies, such as 0.25 and 1 Hz, and the modulation of sleep oscillations, including delta and sigma activity, by binaural beats. In conclusion, this study demonstrated the potential of binaural beats at slow frequencies, specifically 0.25 Hz, for inducing slow-wave sleep in generally healthy populations.

**Supplementary Information:**

The online version contains supplementary material available at 10.1038/s41598-024-76059-9.

## Introduction

Humans spend approximately one-third of their lifespans sleeping. Sleep cycles comprise two distinct states: rapid eye movement (REM) and non-REM (NREM). According to the American Academy of Sleep Medicine (AASM) criteria, NREM sleep can be subclassified into three different electroencephalographic patterns, namely N1, N2, and N3^1^. N3 sleep, also known as slow-wave sleep (SWS), is characterized by the presence of slow waves on electroencephalography (EEG), with at least 20% of a 30-s epoch containing these slow waves, according to the AASM criteria. SWS is necessary for maintaining general health owing to its effects on a variety of physiological and psychological processes, including glucose metabolism, hormone release, immunity, and memory^2^. Nevertheless, stressors associated with modern lifestyles may contribute to SWS disruption^3^, particularly in industrialized nations such as the US and Japan^4,5^. Pharmacological interventions for improving sleep are available; however, many have adverse effects and carry risks of tolerance and dependence. Nonpharmacological treatments such as cognitive behavioral therapy are effective; however, they are expensive, not readily accessible, and associated with high dropout rates^6^. Thus, there is a pressing need to develop convenient and cost-effective nonpharmacological treatments that improve sleep.

One such treatment is binaural beats (BBs), a type of auditory intervention for sleep^7^ that has been described as a “digital drug”^8^. BBs occur when two tones with slightly different frequencies are transmitted separately to both ears, and a single oscillating signal is perceived at a frequency equal to the frequency difference^9^. The perception of BBs is facilitated by a key neural mechanism involved in sound localization^10^. The experience of BBs varies depending on the frequency difference between the binaural oscillating signals, ranging from a rotating tone moving from one ear to the other and back (particularly when the frequency difference is < 1 Hz), to a beating tone characterized by periodic fluctuations in loudness, or even a fast-beating tone producing a sensation of roughness^11–13^. Despite the varying perceptions and individual differences in perception, BB stimulation is a convenient and popular method to modify brain states and functions. However, its efficacy remains to be scientifically verified^14^.

The efficacy of BB stimulation on sleep states and functions has not yet been established. Several studies investigating BBs in the delta band (0.5–4 Hz) revealed promising effects on sleep^15–18^ and sleep oscillations^15,18,19^. However, these studies had limitations and demonstrated incomplete effects. For example, Jirakittayakorn & Wongsawat (2018) applied BBs at a frequency of 3 Hz after the onset of N2, resulting in several effects, such as a reduction in N3 latency and N2 duration and an increase in N3 duration^15^. However, the effects of BB before N2, such as on latencies to N1 and N2, were not demonstrated. Takahara and Horikoshi (2018) found that 1-Hz BBs could increase SWS, albeit with a small sample population of six male participants, which weakens the reliability of the results^18^. A more recent study demonstrated that 1-Hz BBs could enhance slow-wave activity. However, this study could not examine the individual effect of 1-Hz BBs on slow-wave sleep because all auditory conditions were applied during the same sleep period^19^. Two studies conducted by the same researcher group found that several mixed BB frequencies in the delta band (0.5, 2, and 4 Hz) resulted in decreased N2 latencies^16,17^. However, the effect of each individual frequency was unclear, particularly that of 0.5 Hz, which belongs to the range of slow oscillations of < 1 Hz.

In humans, slow oscillations (< 1 Hz) form the hallmark of EEG activity during SWS, organize sleep oscillations such as spindles and delta waves^20^, and play a key role in memory consolidation^21^. Modulation of slow oscillations may affect sleep and post-sleep performance. Previous studies have used various slow rhythmic stimulations (< 1 Hz) to modulate sleep and sleep oscillations^22–30^, with effects including increased SWS, improved memory performance, enhanced slow oscillation activity, and entrainment of sleep oscillations. These stimulations are hypothesized to influence sleep oscillations via neural entrainment, a process whereby intrinsic neural oscillations in the brain synchronize with external and internal forces oscillating at certain frequencies^31^. However, whether < 1 Hz BBs can entrain slow oscillations at the same frequencies and consequently modulate SWS remains unknown.

Previous studies have shown that slow oscillations during sleep are distributed across frequencies of < 1 Hz. Particularly, slow oscillations during slow-wave sleep peak at 0.8 Hz^32–34^. Certain previous studies have used this frequency range for slow rhythmic stimulation^28–30^. However, a smaller peak was also found at approximately 0.25 Hz, indicating the potential existence of a class of even slower oscillations^34,35^. This class of slow oscillations may differ in amplitude, frequency, and function from the typically defined delta range (0.5–4 Hz)^35,36^. For example, ~ 0.3 Hz slow oscillations are proposed to group spindles and delta waves^35^. The distinction between delta waves and slow oscillations at approximately 0.25 Hz may be even more pronounced than that between delta waves and slow oscillations at approximately 0.8 Hz, which overlap with the delta range and have been the focus of many studies. Few studies have specifically investigated the modulation of slow oscillations at approximately 0.25 Hz; nevertheless, existing research, including studies using rhythmic rocking, supports the effectiveness of this frequency in enhancing slow oscillations^22,23^ and improving sleep. Sleep improvements include reduced N2^22,23^ or N3 latency^25^ and increased N2^23,26^, N3^22^, or combined N2 + N3 duration^24^. The underlying mechanism may involve neural entrainment of slow oscillations by 0.25-Hz stimulation^22^. According to this hypothesis, stimulations other than rocking, such as BB stimulation in the proximity of 0.25 Hz, should yield similar effects.

Therefore, this study aimed to investigate whether slow BB stimulation at 0.25 Hz can entrain slow oscillations in the brain and subsequently modulate SWS. The novelty of this study lies in applying a different frequency and elucidating a particular class of slow oscillations, including the role in sleep function and underlying neural entrainment response to stimulation at approximately 0.25 Hz. We hypothesized that BBs at slow frequencies, such as 0.25 Hz, can entrain slow oscillations at the corresponding frequencies. Consequently, we proposed that BBs can modulate key sleep oscillations, such as delta and sigma (11–16 Hz) oscillations, to enhance SWS by shortening the latency to enter SWS and/or increasing its duration. Furthermore, we attempted to replicate the findings of a previous study^18^ using 1-Hz BBs for the following reasons. First, 1 Hz is currently the lowest frequency of BBs used for sleep intervention, lying on the edge of slow frequencies. Second, although previous research has suggested that 1-Hz BBs can increase SWS, the results were derived from a preliminary study with a small sample of only six male participants. Thus, the effect of 1-Hz BBs on sleep requires further confirmation.

## Methods

### Participants

Twelve healthy adults (six women; mean age: 25.4 ± 2.6 years; see Table 1 for characteristics) were deemed to be eligible for participation in this study after screening. The number of participants was determined based on the following considerations: each participant would be subjected to a total of four experiments comprising one sham condition (without auditory stimulation) and three BB conditions, namely 250–250 Hz (0-Hz BB condition), 250–250.25 Hz (0.25-Hz BB condition), and 250–251 Hz (1-Hz BB condition). We chose 250 Hz as the carrier frequency simply because of a major reference study used this frequency, rather than due to any specific theoretical or empirical justification^15^. Some studies have suggested and adopted a carrier frequency of 400 Hz for better effects^12,37^, whereas others used 250 Hz^15,38^. In this study, we tested BBs based on the 250 Hz carrier frequency, ensuring that all included participants could perceive them. To account for the potential effects of the BB condition order, the sequence was assigned using the Latin square design method.

**Table 1:** Demographic characteristics of the participants (*N* = 12).

Characteristics	
Age (Mean +/- SD), years	25.4 +/- 2.6
Sex	
Male	6
Female	6
BMI (mean +/- SD), kg/m^2^	21.3 +/- 1.9
MEQ (mean +/- SD), points	53.2 +/- 4.7
MEQ—Chronotype	
Morning	2
Intermediate	10
PSQI (mean +/- SD), points	3.3 +/- 1.6

Note: BMI, body mass index; MEQ, Morningness-Eveningness Questionnaire; PSQI, Pittsburgh Sleep Quality Index.

A correlation study on the induction of SWS using pink noise at 0.8 Hz for a 90-min daytime nap included 10 participants^30^. Therefore, we used this sample size as a reference. We set the target sample size at 12 to ensure a minimum of 10 participants and equalize the effect of the intervention sequence (a multiple of 4).

The inclusion criteria for this study were as follows: (1) aged between 20 and 60 years; (2) ability to fill out the Japanese instruction documents, consent forms, and survey forms; (3) ability to sleep in examination rooms in the sleep laboratory; (4) not undergoing treatment for any sleep disorder; and (5) without hearing impairments. The exclusion criteria for participation were as follows: (1) irregular sleep/wake cycles (regular: sleep time between 9:00 pm and 1:00 am, wake time between 6:00 am and 9:00 am; 7–9 h of sleep), or history of sleep disorders; (2) body mass index (BMI) < 18.5 kg/m^2^ or > 25 kg/m^2^; (3) habitual smoking; (4) regular alcohol consumption (at least 40 g of pure alcohol twice a week or more); (5) > 300 mg of caffeine consumption daily; (6) engagement in night-shift work after 10 pm within the last three months; (7) traveled to a country outside of Japan with a time difference of 3 h or more within the last three months; (8) a score of ≥ 5.5 on the Pittsburgh Sleep Quality Index (PSQI)^39^; (9) a score of ≤ 30 (extreme evening type) or ≥ 70 (extreme morning type) on the Morningness-Eveningness Questionnaire (MEQ)^40^; (10) the presence of claustrophobia; (11) pregnancy or the possibility of pregnancy; (12) lactation; (13) unstable medical or psychiatric conditions; and (14) feeling of discomfort when listening to the auditory stimulation samples of this experiment or low ability to discriminate BBs (see the screening section).

The experimental protocol was approved by the Clinical Research Ethics Review Committee of the University of Tsukuba Hospital (R02-214) and pre-registered in the University Hospital Medical Information Network Clinical Trials Registry (trial ID: UMIN000042691, dated 12/08/2020). We confirm that all experiments were performed in accordance with the relevant guidelines and regulations. All participants were recruited from the public and provided written informed consent. The study’s purpose and experimental procedures were explained to all participants before study initiation, except for blinding to the order of the auditory stimuli delivered on each experimental day.

### Stimuli

Three acoustic stimuli were utilized in this study: (1) 0.25-Hz BBs with a 250-Hz carrier tone (a pure 250-Hz tone was presented to the left ear and a pure 250.25-Hz tone to the right ear), (2) 1-Hz BBs with a 250-Hz carrier tone (a pure 250-Hz tone was presented to the left ear and a pure 251-Hz tone to the right ear), and (3) 0-Hz BBs with 250-Hz pure tones was delivered to both ears. These acoustic stimuli were explicitly generated by MATLAB 2019b (The MathWorks, Inc., Natick, MA, USA) and delivered to the participants via wired earphones (TaoTronics TT-EP002JP, TaoTronics, Sunvalley Group, Shenzhen, People’s Republic of China), with an extension cord connected to a ZOOM UAC-2 audio interface (ZOOM CORPORATION, Tokyo, Japan). These acoustic stimuli were set to a 60-dB sound pressure level (SPL), in accordance with a previous study^30^. The SPL was calibrated before each experiment using ear simulators housed within a dummy head with integrated ear models (Type 2128E, SAMURA HATS 3500, and Type 4565/4566; SOUTHERN ACOUSTICS Co., Ltd., Kamakura, Japan) to ensure high-precision acoustic measurement.

### Screening

Before the experiments commenced, all participants mandatorily underwent two screening procedures to confirm that they fulfilled the eligibility criteria. The first step of the online screening involved obtaining the participants’ self-reported BMI, PSQI, and MEQ scores. Only those who passed the first online screening were invited to attend the second screening in the experiment room. The second screening was conducted to measure the participants’ actual BMI, and test their pleasantness with and perception of the BBs used in this study. The pleasantness test was designed to ensure that participants would not experience discomfort with the 0-Hz, 0.25-Hz, and 1-Hz BBs. The test for perceptual ability constituted two sessions. One session was designed to test the perception of 1-Hz BBs and the other for the perception of 0.25-Hz BBs.

During the second screening, participants listened to each type of BBs for 5 min and rated the pleasantness as follows: 1, very unpleasant; 3, unpleasant; 5, neither unpleasant nor pleasant; 7, pleasant; and 9, very pleasant. Participants with a rating of 5 or higher or those with a rating of 4 but were confirmed not to be hindered by BBs in participating in the experiments were included in the subsequent discrimination test for participation. Our preliminary studies indicated that not all participants could perceive the BBs, leading to concerns regarding the potential impact on BB efficacy. We chose to include only participants who could discriminate between the different frequencies of BBs used in the current study to ensure the integrity of our research findings.

The discrimination test constituted forty trials divided into two sessions (twenty trials per session). One session was designed to test the perception of 1-Hz BBs and the other for the perception of 0.25-Hz BBs, with a 10-min break between the two sessions. Each trial constituted two 12-s auditory stimulus samples with a 6-s interval and approximately 15 s to answer the questionnaire about whether the two auditory stimulus samples were the same. Table S1 presents the combination list of the two auditory stimuli in every trial. Participants whose accuracy and F-scores exceeded 80% in each session were considered to have passed the test. The accuracy and F-score were expressed explicitly as follows:

*Accuracy* = (TP + TN) / (TP + TN + FN + FP).

*Precision* = TP / (TP + FP).

*Recall* = TP / (TP + FN).

*F − score* = 2 × Precision × Recall / (Precision + Recall),

where TP is true positive, FP is false positive, TN is true negative, and FN is false negative.

### Experimental protocol

The experimental protocol was conducted over four days for each participant, corresponding to the sham condition (no acoustic stimulation during the 90-min nap) and the 0-Hz, 0.25-Hz, and 1-Hz BB conditions. The order of experimental conditions was counterbalanced across participants based on the Latin square method. Data from each participant were recorded using sleep diaries and a wearable sleep tracker (Fitbit Charge 3, Fitbit Inc., San Francisco, CA, USA) for the seven preceding nights before each experimental day. The time between experimental days varied from 7 to 46 days, with a median of 12 days. The average inter-experimental period across all participants was 14.42 +/- 6.60 days. This variation allowed us to accommodate participants’ schedules while ensuring sufficient rest between experiments, thus avoiding carry-over effects.

Each participant arrived at the experiment room around noon on all experimental days. We provided the participants with lunch and controlled their caloric intake based on their weight. Lunchtime lasted until 12:40 pm. The pre-nap task session commenced at 12:45 pm. First, we administered the 10-min Psychomotor Vigilance Test (PVT) to objectively measure behavioral alertness^41^. Thereafter, participants completed the Japanese version of the Karolinska Sleepiness Scale (KSS-J)^42^ by self-rating their sleepiness on a 9-point scale (1, very awake to 9, very sleepy) and the Profile of Mood States 2nd Edition-Adult Short (POMS2)^43,44^ by self-rating their mood on a 5-point scale (0, not at all to 4, very much) to measure subjective sleepiness and mood states. Participants were fitted with polysomnography (PSG) and other devices, including earphones, from 1:00 pm to 2:25 pm. Acoustic stimulations with the SPL calibrated using the above-mentioned system were delivered through in-ear headphones starting 2 min before lights out at 2:30 pm and continuing throughout the entire nap period. Participants were allowed a 90-min nap (from 2:30 to 4:00 pm) in a sound-proof chamber. No auditory stimulation was present during the 90-min nap in the sham condition. However, 250-Hz pure tones were presented to the participants’ ears for only 2 min before the nap period (from 2:28 to 2:30 pm), just before lights off, to maintain sham blinding. In the other conditions, the corresponding auditory stimuli were delivered to participants starting 2 min before the nap (2:28 pm) and continuing during the 90-min nap session. The PSG equipment was removed after lights on at 4:00 pm, and the same tasks performed before the nap were resumed from 4:30 pm.

Each participant was instructed to lie on a comfortable bed in a sound-proof chamber during the 90-min nap session. Meanwhile, Polymate Pro MP6100 (Miyuki Giken Co., Ltd., Tokyo, Japan), designed to measure the direct current (DC) component of an EEG signal, recorded the PSG data. This device excels in capturing infra-slow oscillations, making it ideal for exploring the slower frequencies associated with brain activity often encountered in sleep studies. With a sampling frequency of 500 Hz, the Polymate Pro MP6100 can capture EEG activity as DC within the frequency range of 167 Hz. The EEG electrodes were positioned based on the standard PSG recording method^1^ and international 10/20 system^45^ as follows: F3, F4, C3, C4, T3, T4, O1, O2, Fz, Cz, Pz, M1, and M2. Moreover, two electrooculography (EOG), three chin electromyography, and two electrocardiography electrodes were attached. Sleep stages were scored in 30-s epochs by a registered polysomnographic technologist (Y.S.) according to the AASM criteria^1^. This study focused on sleep parameters, including total sleep time (TST), sleep efficiency, sleep latency (SL), arousal index, wake after sleep onset, duration of each sleep stage, percentage of TST in each stage, N2 latency, N3 latency, REM latency, and arousal index (ArI).

### EEG power spectrum analysis

Fast Fourier transform (FFT) analysis was conducted based on two different epoch lengths of 30 s and 4 s. The 30-s FFT analysis was used to investigate whether BBs at or around slow frequencies ($\:\le\:$ 1 Hz) can entrain neural oscillations at the corresponding frequencies. On the other hand, the 4-s FFT analysis was used to investigate the modulation of sleep oscillations, such as delta and sigma oscillations.

Before the FFT analysis, raw EEG data were preprocessed by a customized program combined with the EEGLAB toolbox^46^ in MATLAB (The MathWorks Inc., Natick, MA, USA). The preprocessing steps were as follows: (i) artifacts arising from body movements and excessive sweating were identified and removed by visual inspection. In addition, one bad channel (T4) in one condition (0.25-Hz BBs) from one participant was removed; (ii) the cleaned data were further preprocessed. First, channel locations were registered; second, all channels were re-referenced to average mastoids (M1, M2); and third, a basic FIR filter in the EEGLAB toolbox was applied between 0.3 and 35 Hz according to the routine presented in the AASM manual^1^; (iii) data from the F3, F4, C3, C4, T3, T4, O1, O2, Fz, Cz, Pz, and two EOG channels were subjected to independent component analysis (ICA) utilizing the default “Infomax runica.m” algorithm^46^. Components with eye-related artifacts were identified if they had a possibility larger than 5%; and (iv) we applied the ICA results back to the pre-filtered data and removed the identified components. This approach allowed us to increase the efficiency of ICA using a narrower bandpass filter (0.3–35 Hz). Moreover, it enabled us to utilize the ICA results and investigate a broader frequency range in the raw data, particularly frequencies < 1 Hz.

Subsequently, with the unfiltered raw data, we calculated the power spectra for 30-s epochs without overlap using the FieldTrip toolbox^47^ for each channel, stimulation condition, and sleep stage. In addition, we calculated the power spectra for 4-s epochs with a 1-s overlap. We segmented each 30-s epoch into overlapping 4-s epochs, resulting in ten 4-s segments per 30-s period. In this manner, the 10th 4-s epoch included 1-s of data beyond the boundary of the corresponding 30-s epoch. Nonetheless, this 10th epoch was classified within the same sleep stage as the corresponding 30-s epoch since a majority of its data is encompassed within the latter. Furthermore, only nine 4-s epochs were considered for the final 30-s sleep epoch, before the lights were turned on. This is because the last, or 10th, 4-s epoch extended 1-s beyond the designated sleep time or occurred after sleep onset.

Before FFT analysis, all epochs were demeaned and detrended. During FFT analysis, we applied the “mtmfft” method with the “hanning” window, choosing a specific, non-2^n padding length (which can be handled by the FieldTrip toolbox), which was four times the epoch length. This was done to increase the frequency resolution to 1/120 Hz for 30-s epochs and 1/16 Hz for 4-s epochs. The relative power for the 30-s and 4-s epochs was derived for further statistical analysis by dividing the absolute power by the sum of the power of the total band, which was 0.125–30 Hz. The lower frequency of 0.125 Hz was selected because it encompasses 0.25 Hz and matches the lowest frequency (1/8 Hz) for both the 30-s (1/120 Hz) and 4-s epochs (1/16 Hz). The upper frequency of 30 Hz was chosen as it represents the upper beta band, which is typically the highest frequency used in sleep research. We calculated the average spectral power by pooling all epochs of the same stage together and computing the mean for 0.25 Hz, 1 Hz, delta band, and sigma band. We calculated the absolute power for these frequencies for reference. Moreover, we calculated the relative power in these bands at 2-min intervals (i.e., averaging the power from every four consecutive 30-s epochs) across three representative electrodes (Fz, Cz, and Pz) over the 90-min nap period, from lights out to lights on, to explore the time course of sleep oscillations in the delta and sigma bands.

### Statistical analysis

A two-tailed Wilcoxon signed-rank test was conducted to determine differences in sleep parameters and EEG power between the BB conditions (0-Hz vs. sham, 0.25-Hz vs. sham, 1-Hz vs. sham, 0.25-Hz vs. 0-Hz, 1-Hz vs. 0-Hz, 1-Hz vs. 0.25-Hz). This was a proof-of-concept study; therefore, the analysis did not correct for type I errors due to multiple comparisons for the BB conditions. On the other hand, to prevent type 1 errors arising from conducting statistical tests across multiple channels simultaneously, EEG power for delta and sigma bands and 0.25 Hz and 1 Hz frequencies were subjected to cluster-based permutation tests in FieldTrip (see https://www.fieldtriptoolbox.org/workshop/madrid2019/tutorial_stats/) for comparisons between two BB conditions. For the time-course analysis of relative power in the delta and sigma bands, a false discovery rate (FDR) correction was applied to control for the increased likelihood of type I errors due to multiple comparisons across time points.

The inverse-transformation of the reaction time of PVT (response speed, sec^−1^), the number of times the reaction time was more than or equal to 500 ms (errors of omission, i.e., lapses), KSS-J scores, and POMS2 scores were analyzed using the Wilcoxon signed-rank test, without corrections for multiple comparisons. When testing the differences between the two sessions, the data collapsed across the BB conditions.

## Results

### Screening

Thirty-nine individuals participated in the first screening step (see Figure S1). Five participants were excluded because their PSQI scores were ≥ 5.5, indicating possible sleep disturbance. Additionally, one participant was excluded because she was underweight (BMI<18.5 kg/m^2^).

Twenty-nine of 33 participants who passed the first screening participated in the second step (four participants quit before this step). One participant was excluded because his actual BMI was ˂18.5 kg/m^2^. The remaining 14 participants were excluded because of low accuracy in the BB discrimination test. Specifically, nine participants failed to discriminate the 0.25-Hz BBs from the 0-Hz BBs, and five failed to discriminate both the 0.25-Hz and 1-Hz BBs from the 0-Hz BBs. Among the 14 excluded participants, six found the BBs unpleasant: two found the 1-Hz BBs unpleasant, one found the 0.25-Hz BBs unpleasant, and three found both unpleasant (scores < 5). Among the remaining 14 participants, two rated the 0.25-Hz BBs and one rated the 0-Hz BBs as 4. However, when asked to confirm if they were uncomfortable, they replied that they had adapted to the stimulus. Thus, they were included in the study. Overall, 14 participants passed this screening session.

The targeted sample size for this study was 12. However, before the completion of all experiments, two participants had missing data for the following reasons: one participant withdrew from the experiments due to personal reasons, and the other participant’s PSG data in the 1-Hz BB condition could not be recorded due to technical issues. Therefore, these two participants were excluded. Thus, although 14 participants passed the screening and agreed to engage in the nap experiments, the final analysis included 12 participants (see Table 2 for the screening results).

**Table 2:** Results of the two-phase screening process.

Parameters	0.25-Hz BBs (%)	1-Hz BBs (%)
1st screening passing rate	84.6	
2nd screening passing rate	50.0	82.1
Accuracy	96.1 ± 5.9	96.4 ± 4.6
F-score	96.0 ± 6.1	96.5 ± 4.2

Note: BBs, binaural beats.

### Sleep parameters

**Figure 1:**
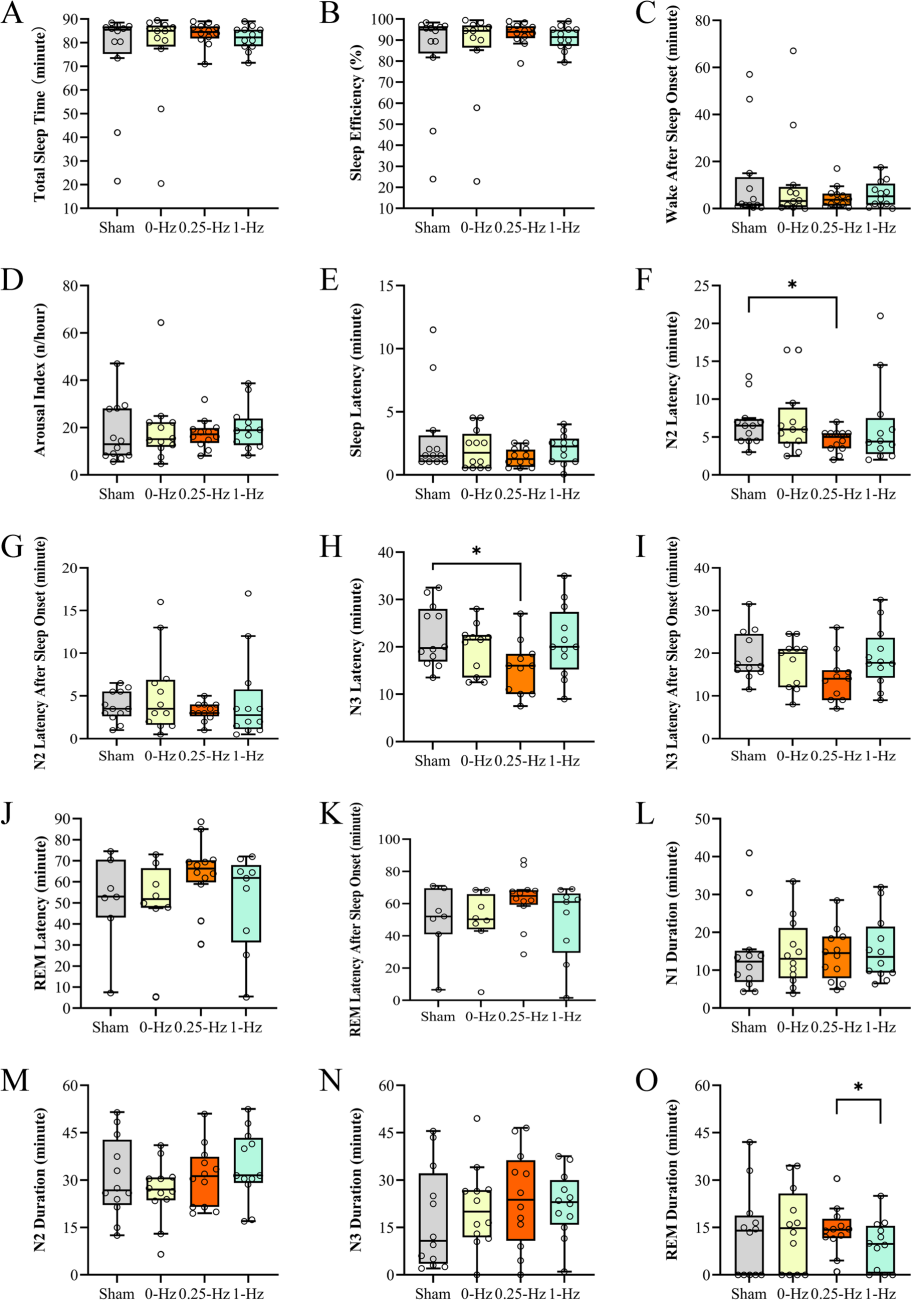
Sleep parameters summarized as (**A**) total sleep time (min), (**B**) sleep efficiency (%), (**C**) wake after sleep onset (min), (**D**) arousal index (n/h), (**E**) sleep latency (min), (**F**) N2 latency (after lights out, min), (**G**) N2 latency after sleep onset (min), (**H**) N3 latency (after lights out, min), (**I**) N3 latency after sleep onset (min), (**J**) rapid eye movement (REM) sleep latency (after lights out, min), (**K**) REM latency after sleep onset (min), (**L**) N1 duration (min), (**M**) N2 duration (min), (**N**) N3 duration (min), and (**O**) REM duration (min). The box plot shows the distribution of the individual data points. The top, bottom, and line in the middle of the box shown in the sequence represent the 75th, 25th, and 50th percentiles, respectively. The whiskers represent the highest and lowest values that were not outliers or extreme. Circles beyond the whiskers correspond to outliers and extreme values. * *p*<0.05.

All sleep parameters in the four conditions are shown in Fig. 1. Significant differences were observed only between the 0.25-Hz BB and sham conditions in the N2 latency (W = 7.5, Z = -2.27, *p =* 0.023; Fig. 1F) and N3 latency (W = 10.5, Z = -2.00, *p =* 0.045; Fig. 1H) and between the 1-Hz and 0.25-Hz BB conditions in the REM phase (W = 8.0, Z = -2.43, *p =* 0.015; Fig. 1O). This result indicated that 0.25-Hz BB stimulation may shorten N2 and N3 latencies compared with sham conditions. The effect size derived by matched-pairs rank-biserial correlation^48^ between the sham and 0.25-Hz BB conditions was calculated as 0.773 for the N2 latency and 0.682 for the N3 latency (Table S2 shows the effect size of Cohen’s *d*). The time difference between the onsets of N2 and N3 was compared between the conditions, and none of the comparisons were significant (all p-values > 0.091).

### Behavioral performance

**Figure 2:**
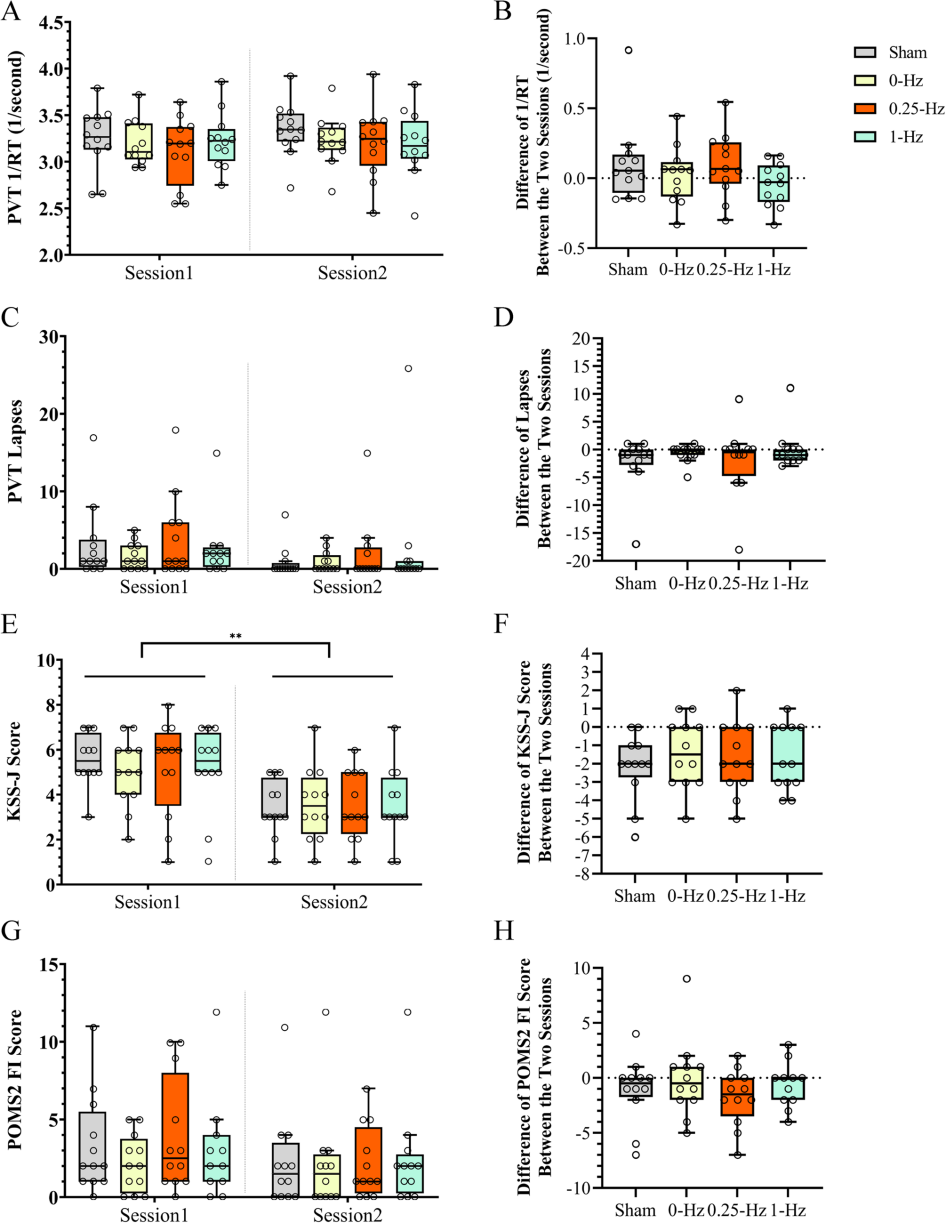
Behavioral performance. Session 1: before-nap sessions, and Session 2: after-nap sessions. (**A**) PVT response speed (1/RT, 1/s) in the two task sessions among the four conditions, (**B**) differences in response speeds in PVT performance between the two task sessions and four conditions (1/RT, 1/s), (**C**)the number of lapses in the PVT in the two task sessions and four conditions, (**D**) differences in the number of lapses in the PVT performance between the two task sessions and four conditions, (**E**) KSS-J scores in the two task sessions for the four conditions, (**F**) differences in the two task sessions’ KSS-J scores in the four conditions, (**G**) POMS2-FI scores in the two task sessions across the four conditions, and (**H**) differences in the two task sessions’ POMS2-FI scores in the four conditions. The box plot shows the distribution of individual data points. The top, bottom, and line in the middle of the box shown in the sequence represent the 75th, 25th, and 50th percentiles, respectively. The whiskers represent the highest and lowest values that are not outliers or extremes. Circles beyond the whiskers correspond to outliers and extreme values. PVT, Psychomotor Vigilance Test; KSS-J, the Japanese version of the Karolinska Sleepiness Scale; FI, fatigue-inertia; POMS2, Profile of Mood States 2nd Edition-Adult Short. ** *p*<0.01.

The results of the PVT response speed (1/RT) and reaction delay (lapses) for the two task sessions before and after the nap are shown in Fig. 2A and C. However, neither showed a significant difference between sessions (*p-values*$\:\ge\:$ 0.056). Furthermore, we tested the difference in the 1/RT and number of lapses of the two sessions between conditions (Fig. 2B and D), without significant differences in these two variables between conditions (*p-values* ≥ 0.182).

The KSS-J and POMS2 fatigue-inertia (FI) scores in the pre- and post- nap task sessions are summarized in Fig. 2E-H (see Figure S2 for the scores of other scales of POMS2). The KSS-J scores differed significantly between the two sessions (W = 1.5, Z = -2.95, *p =* 0.003; Fig. 2E), but no significant difference was observed in the POMS2 fatigue scores (W = 20, Z = -1.17, *p =* 0.243; Fig. 2G). Participants’ subjective sleepiness significantly decreased after the nap. However, the respective differences in the KSS-J scores and POMS2 fatigue scores between the two task sessions did not differ significantly between the conditions (*p-values* ≥ 0.165; Fig. 2F and H).

### EEG power

**Figure 3:**
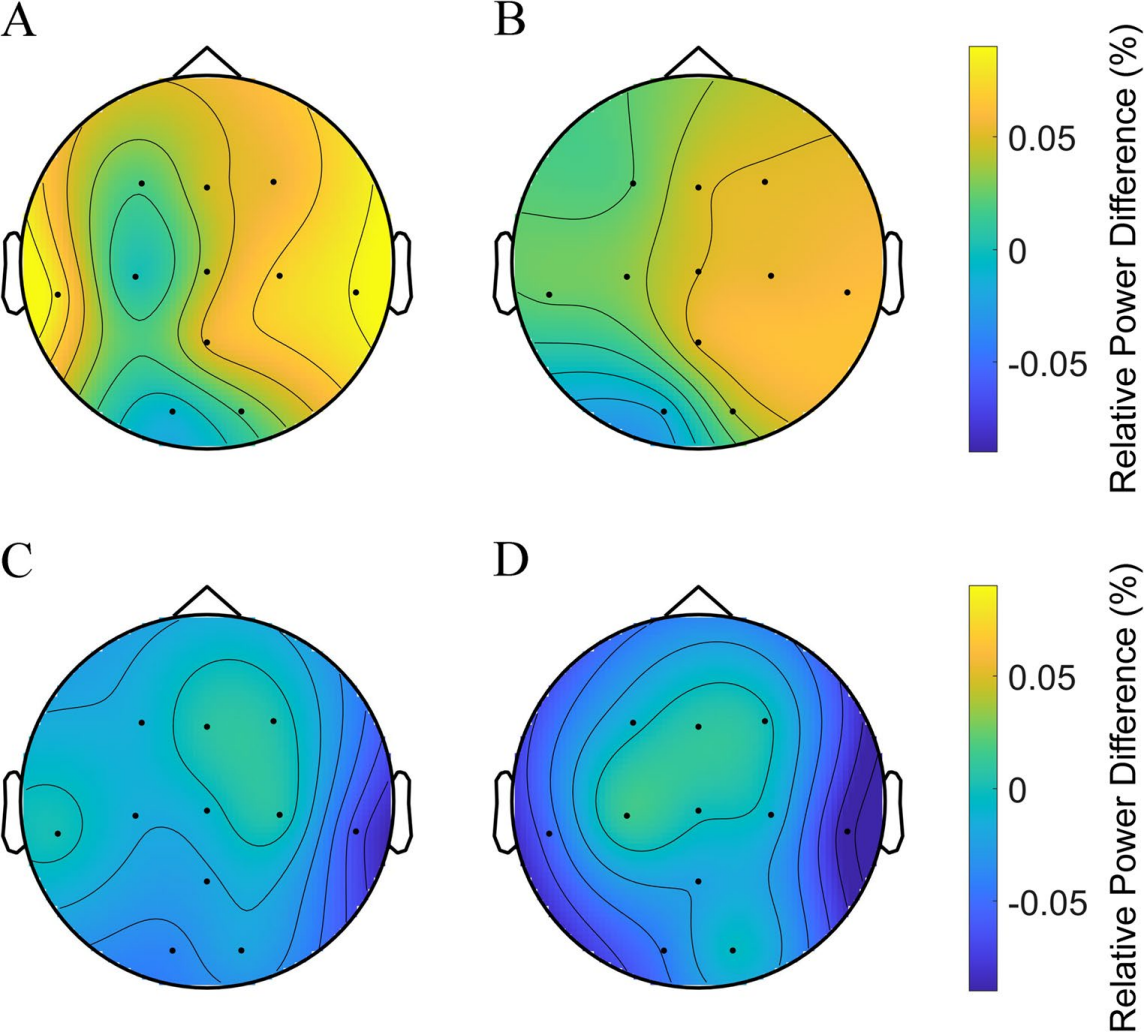
Topographic map illustrating the comparisons of relative delta power for four pairs: (**A**) 0.25-Hz BB vs. the sham, (**B**) 0.25-Hz BB vs. 0-Hz BB, (**C**) 1-Hz BB vs. the sham, and (**D**) 1-Hz BB vs. 0-Hz BB conditions. Dots represent electrodes indicating differences in relative delta power (*p* > 0.05 for black dots, cluster-based permutation test controlling for multiple comparisons). Color intensity reflects the magnitude of differences: yellow signifies a positive difference, whereas blue denotes a negative difference.

**Figure 4:**
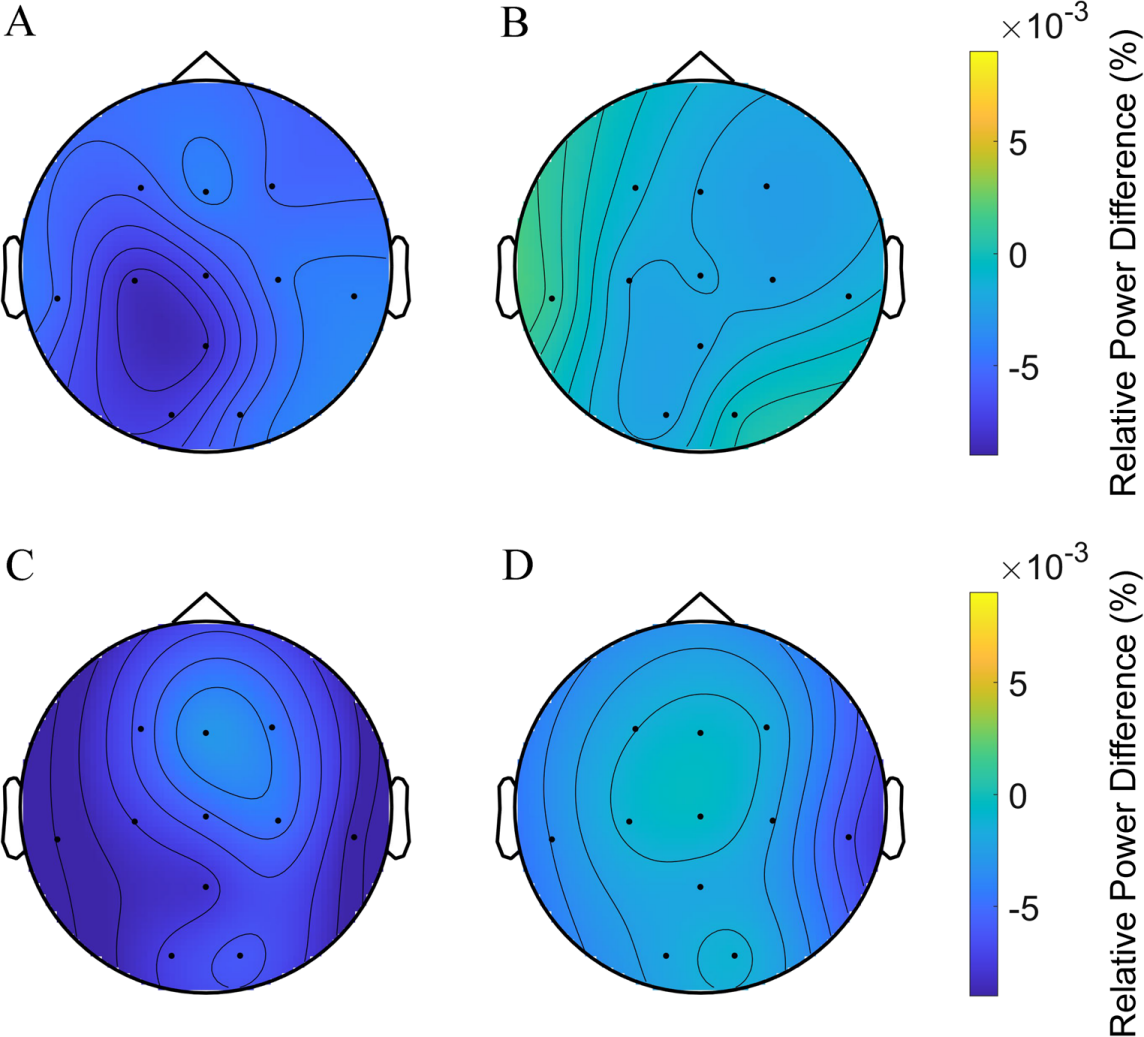
Topographic map illustrating the comparisons of relative sigma power for four pairs: (**A**) 0.25-Hz BB vs. the sham, (**B**) 0.25-Hz BB vs. 0-Hz BB, (**C**) 1-Hz BB vs. the sham, and (**D**) 1-Hz BB vs. 0-Hz BB conditions. Dots represent electrodes indicating differences in relative sigma power (*p* > 0.05 for black dots, cluster-based permutation test controlling for multiple comparisons). Color intensity reflects the magnitude of differences: yellow signifies a positive difference, whereas blue denotes a negative difference.

Considering the effects of BBs on sleep-related brain activity, we compared delta and sigma oscillations during N2/N3 between the conditions. The topographic map in Fig. 3 shows that 0.25-Hz BBs may induce higher relative delta power than the sham and 0-Hz BB conditions across channels. However, the cluster-based permutation test did not yield any significant comparisons. Figure 4 shows that 0.25-Hz BBs may possibly lead to lower relative sigma power than the sham and 0-Hz BB conditions. However, a similar permutation test demonstrated no significant differences. In contrast, 1-Hz BBs led to a less clear trend.

**Figure 5:**
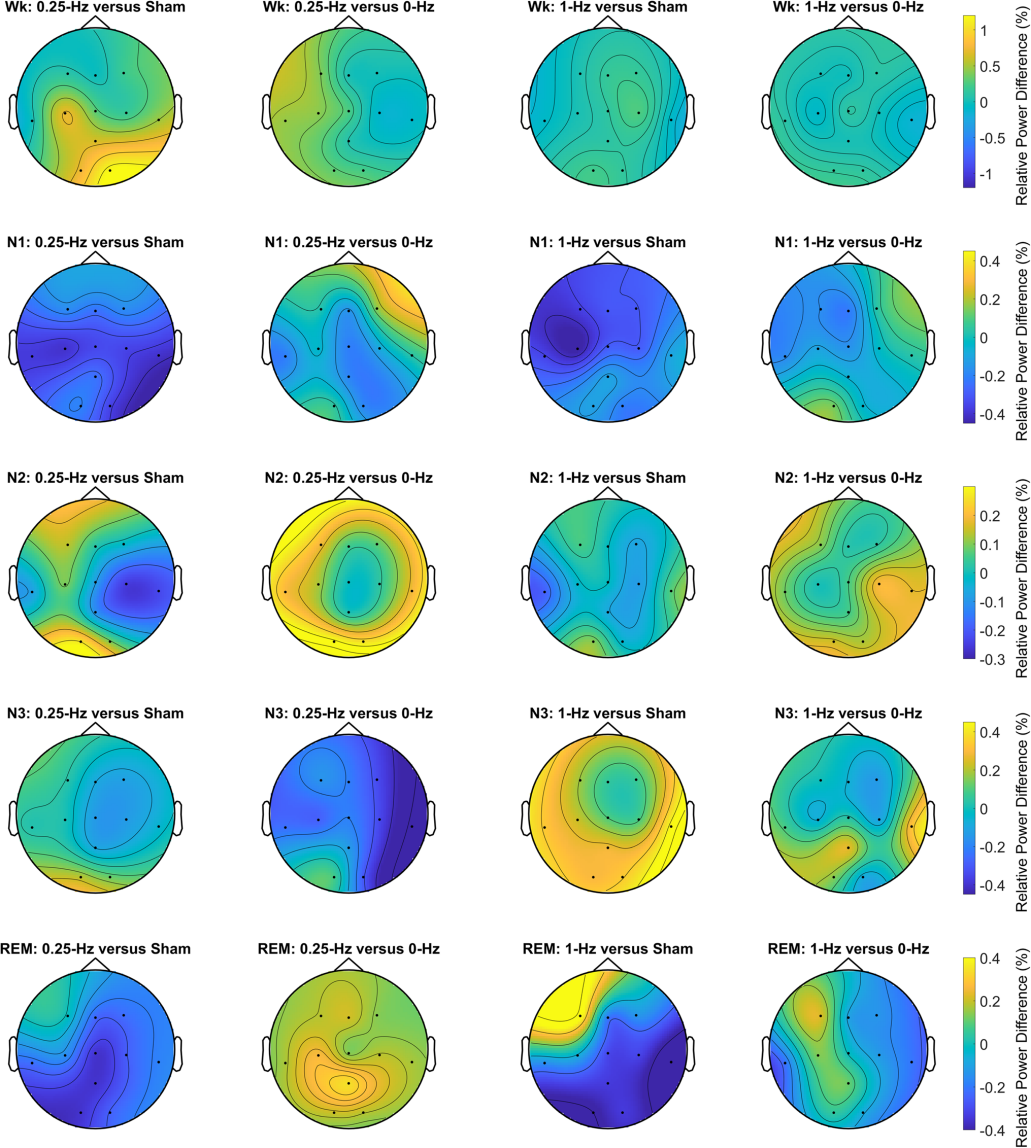
Topographic map illustrating the comparisons of relative power at 0.25 Hz for four pairs: 0.25-Hz BB vs. the sham, 0.25-Hz BB vs. 0-Hz BB, 1-Hz BB vs. the sham, and 1-Hz BB vs. 0-Hz BB conditions during five sleep stages: Wk, N1, N2, N3, and REM. The rows represent the sleep stages, whereas the columns correspond to the four pairs of comparisons. Dots represent electrodes indicating differences in relative 0.25 Hz power (*p* > 0.05 for black dots, cluster-based permutation test controlling for multiple comparisons). Color intensity reflects the magnitude of differences: yellow signifies a positive difference, whereas blue denotes a negative difference.

The relative spectral powers at 0.25 Hz and 1 Hz were extracted and compared between the conditions to obtain evidence for neural entrainment by 0.25-Hz and 1-Hz BBs. Figure 5 shows the topographic maps for comparisons of relative power at 0.25 Hz between the 0.25-Hz BB and sham; 0.25-Hz BB and 0-Hz BB; 1-Hz BB and sham; and 1-Hz BB and 0-Hz BB conditions. We expected that 0.25-Hz BBs would entrain neural oscillations at 0.25 Hz, leading to a higher 0.25-Hz power than the sham or 0-Hz BB conditions. We observed a trend indicating that the relative spectral power at 0.25 Hz might be higher under the 0.25-Hz BB condition than under the sham or 0-Hz BB conditions, particularly in the frontal area during earlier sleep stages (e.g., N2). However, the cluster-based permutation test did not yield significant results. Similarly, we expected but did not observe the entrainment effects of 1-Hz BBs (see Figure S3). We provided the results of absolute power for the delta band, sigma band, 0.25 Hz, and 1 Hz for reference (see Figures S4-7).

In addition, the time-course analysis of relative power in the delta and sigma bands did not show any significant differences between the BB conditions at any time points (see Figure S8).

## Discussion

This exploratory study found novel preliminary evidence on the efficacy of BBs at a slow frequency of < 1 Hz for inducing SWS, specifically at 0.25 Hz on a 250-Hz carrier tone. The results showed that 0.25-Hz BBs notably shortened both N2 and N3 latencies compared to the sham condition. Despite similar post-sleep improvements in PVT performance and reductions in sleepiness across conditions, our study introduces discussions on the mechanism by which BBs affect slow oscillations and other sleep-related activities, including delta and sigma oscillations, suggesting the need for further evidence.

Delta BBs (< 4 Hz) are typically used to modulate SWS and have demonstrated enhancements in sleep quality and EEG parameters^15–17,49,50^; however, research specifically focusing on BBs’ sleep-promotion effects at slow oscillation frequencies (< 1 Hz) is scarce. Only a few studies have explored low-frequency combinations within the delta band (e.g., 0.5, 2, and 4 Hz)^16,17^. This study marks a novel inquiry into the effects of BBs at a singular slow frequency (0.25 Hz) and contributes additional evidence to the potential efficacy of delta/slow frequency BBs in promoting SWS.

Several possible mechanisms can explain the sleep-promotion effects of 0.25-Hz BBs. One mechanism is based on the mood-soothing effects of BB stimulation. BBs are popularly claimed to alter conscious states, such as wake and sleep, and mood; therefore, they have been referred to as a digital drug for these effects^8^, although most of these seemingly “wonderful” effects remain to be verified. Specifically, theta or delta BBs were found to exert beneficial effects on the emotional experience accompanying sleep, such as reducing anxiety, anger^49^, and pain^51^ and increasing parasympathetic activity in the autonomic nervous system^52,53^. However, no scientific evidence supports a causal link between BBs’ effects on mood and sleep. Our findings also did not reveal any significant mood-altering effects of BBs that could influence sleep (see Figure S2). Therefore, the mood-soothing mechanism remains to be confirmed.

Another possible mechanism may be sensory mismatch. For example, sensory mismatch is a possible mechanism for the sleep-promoting effects of vestibular stimulation during rocking movements. Here, sensory mismatch refers to the mismatch between the vestibular sensation of rotation and the translational movement perceived by other senses. This mismatch may cause dizziness and drowsiness, possibly leading to sleep promotion^54^. BB stimulation has been revealed to cause dizziness or discomfort in some cases^55–58^. In our study, slow BBs at 0.25 Hz may have been perceived as a rotating tone moving from one ear to the other and back. A similar sensory mismatch could occur due to the auditory sensation of a rotating sound without a corresponding visual perception of a rotating sound source. In other words, the participant perceives the sound as rotating, even without visual confirmation of the sound source moving, which may create a sensory disconnect. However, most participants (10/12) in our study found the BBs to be neutral to pleasant. The remaining two participants reported slight discomfort but indicated no reluctance to participate in the experiments. Thus, the sensory mismatch hypothesis might not apply to our study.

Neural entrainment may also be a mechanism by which slow BBs induce faster SWS. A recent review suggested that neural entrainment may provide a basis for BBs’ effects on brain states^38^. Previous studies have provided evidence for BB entrainment of neural oscillations at approximately 1 Hz during sleep^19^. However, our study did not yield significant results supporting neural entrainment for frequencies ≤ 1 Hz. If slow oscillations were entrained, other sleep oscillations such as delta and sigma should also be modulated by slow BBs. However, our study revealed no significant results, indicating the need for further investigation.

Unlike 0.25-Hz BBs, 1-Hz BBs did not exert any significant effects on sleep parameters in our study. No evidence supports that neural entrainment is the mechanism by which 0.25-Hz BBs shorten N2/N3 latencies. However, if this hypothesis is correct, neural entrainment can explain this discrepancy. BBs of < 1 Hz may lead to the entrainment of slow oscillations^22^ which may in turn influence other major oscillations associated with SWS, such as delta and sigma (reflecting spindle activity) oscillations^20,59^. However, $\:\ge\:$1 Hz BBs may only modulate delta waves. Notably, slow oscillations (< 1 Hz) and delta waves differ in many aspects, such as their underlying mechanisms^20,60^ and roles in cognitive functions including memory^36^.Entrainment of these two bands of neural oscillations may result in different effects. Moreover, 1 Hz is around the boundary between slow oscillations (< 1 Hz) and delta waves (1–4 Hz)^36,61^. Therefore, BB stimulation at 1 Hz may have induced more complex processes in our study and resulted in the discrepancy between the effects of 0.25-Hz and 1-Hz BBs on sleep.

In addition, our study did not show significant differences in the effects of BB stimulation on PVT performance and fatigue between conditions. The lack of difference in PVT performance suggests that acoustic-BB stimulation did not induce significant cognitive impairment in sustained attention. Moreover, fatigue did not differ significantly between the conditions, suggesting the absence of detrimental effects of slow BBs on subjective experiences in this study.

Thus, slow BB stimulation at 0.25 Hz holds the potential to facilitate SWS. Specifically, 0.25 Hz BBs on a 250 Hz carrier tone can be utilized as a stimulus for inducing faster SWS in various situations such as napping in the office, sleeping in different time zones, and in those with insomnia. BBs can be integrated within music or autonomous sensory meridian responses, as shown in previous studies^16,58,62^, potentially serving as one of the most promising, safe, convenient, cost-effective, and easily accessible non-pharmacological treatments for improving sleep.

This study had some limitations. First, this was an exploratory study with a small sample size. Therefore, a non-parametric statistical analysis was used, without multiple comparison corrections for the BB conditions. Second, the participants were young healthy adults. The effects of BBs on the sleep of elderly participants require further validation. Moreover, determining whether populations with sleep disorders, such as insomnia, can benefit from BB stimulation is important. Our next study will target subclinical participants with insomnia symptoms (trial ID: UMIN000048122, dated 06/25/2022), aiming to provide novel insights into this issue. Third, we found that several participants had difficulty perceiving the BBs. Specifically, discriminating BBs between 0.25-Hz and 0-Hz was difficult. We only included participants who could distinguish between BBs of different frequencies at a high standard. Future studies should investigate whether individuals who have difficulty perceiving BBs can still benefit from BB stimulation. Intriguingly, our next study (trial ID: UMIN000048290, dated 07/09/2022) will specifically recruit participants who are unable to discriminate between the BBs to further explore the significance of BB perception. Fourth, we did not control for menstrual cycle phases or the use of hormonal contraceptives among female participants. Future studies should consider these confounding factors, which may impact sleep patterns. Fifth, the 250-Hz tone was consistently delivered to the left ear, whereas tones of different frequencies were delivered to the right ear to create BB perception. Whether reversing laterality while delivering auditory stimulation would yield the same results remains unknown. In addition, some studies have suggested that a 400-Hz tone may be a better carrier, a finding requiring further confirmation to enable future selection of BB carriers. Sixth, our study applied a daytime nap protocol. Future studies should validate our results during nighttime sleep.

In conclusion, to the best of our knowledge, this study is the first to examine the effects of BBs at frequencies as low as 0.25 Hz on sleep architecture during a 90-min daytime nap. This study provided preliminary evidence that listening to slow BBs may enhance the transition from wakefulness to N2 and N3 sleep. Our study provides insight into potential novel non-pharmacological and non-invasive methods to facilitate SWS initiation. Further studies with a sufficient sample size are warranted to confirm the findings of this study. Moreover, the generalizability of the BB effect in promoting SWS should be investigated in broader populations, such as individuals who cannot precisely perceive BBs, the elderly, and patients with sleep disorders such as insomnia.

## Supplementary Information

Supplementary Material 1.

## Data Availability

The data in this article will be shared upon reasonable request to the corresponding author.
