# Supplementary material for: Binaural beats at 0.25 Hz shorten the latency to slow-wave sleep during daytime naps
Source: Sci Rep. 2024 Oct 30;14:26062. doi: 10.1038/s41598-024-76059-9 (PMC11525714; doi:10.1038/s41598-024-76059-9)
Supplement: Supplementary file 1 — Supplementary Material 1. [file 41598_2024_76059_MOESM1_ESM.docx]

Binaural beats at 0.25 Hz shorten the latency to slow-wave sleep during daytime naps

**Zhiwei Fan^1,2,†^, Yunyao Zhu^1,3,†^, Chihiro Suzuki^1^, Yoko Suzuki^1^, Yumi Watanabe^4^, Takahiro Watanabe^4^, and Takashi Abe^1,*^**

^1^ International Institute for Integrative Sleep Medicine (WPI-IIIS), University of Tsukuba, 1-1-1 Tennodai, Tsukuba, Ibaraki 305–8575, Japan

^2^ The Japan Society for the Promotion of Science (JSPS) Foreign Researcher, Tokyo, Japan

^3^ Graduate School of Comprehensive Human Science, University of Tsukuba, Tsukuba, Japan

^4^ KYOCERA Corporation, Kyoto, Japan

^†^ Co-first authors

* **Corresponding author**: Takashi Abe

International Institute for Integrative Sleep Medicine (WPI-IIIS), University of Tsukuba, 1-1-1 Tennodai, Tsukuba, Ibaraki 305–8575, Japan

Email: abe.takashi.gp@u.tsukuba.ac.jp

Institution where work was performed: International Institute for Integrative Sleep Medicine (WPI-IIIS), University of Tsukuba, 1-1-1 Tennodai, Tsukuba, Ibaraki 305–8575, Japan

**Table S1.** Combination list of the two auditory stimuli in every trial during the two screening sessions

| Session 1 | Session 2 | Number of trials |
| --- | --- | --- |
| S1–S1 | S1–S1 | 5 |
| S1–S2 | S1–S3 | 5 |
| S2–S2 | S3–S3 | 5 |
| S2–S1 | S3–S1 | 5 |

Note: S1 represents the 0-Hz binaural beats (BBs); S2 represents the 0.25-Hz BBs; S3 represents the 1-Hz BBs

.

**Table S2.** Effect size calculation

|  |  | 0.25-Hz BBs vs. Sham | |  | 0.25-Hz vs. 0-Hz BBs | |
| --- | --- | --- | --- | --- | --- | --- |
| Parameters |  | N | Cohen's d |  | N | Cohen's d |
| N2 Latency |  | 12 | 0.788 |  | 12 | 0.557 |
| N3 Latency |  | 11 | 0.727 |  | 10 | 0.443 |

Note: BBs represent binaural beats.





**Figure S1**. Flow diagram of screening


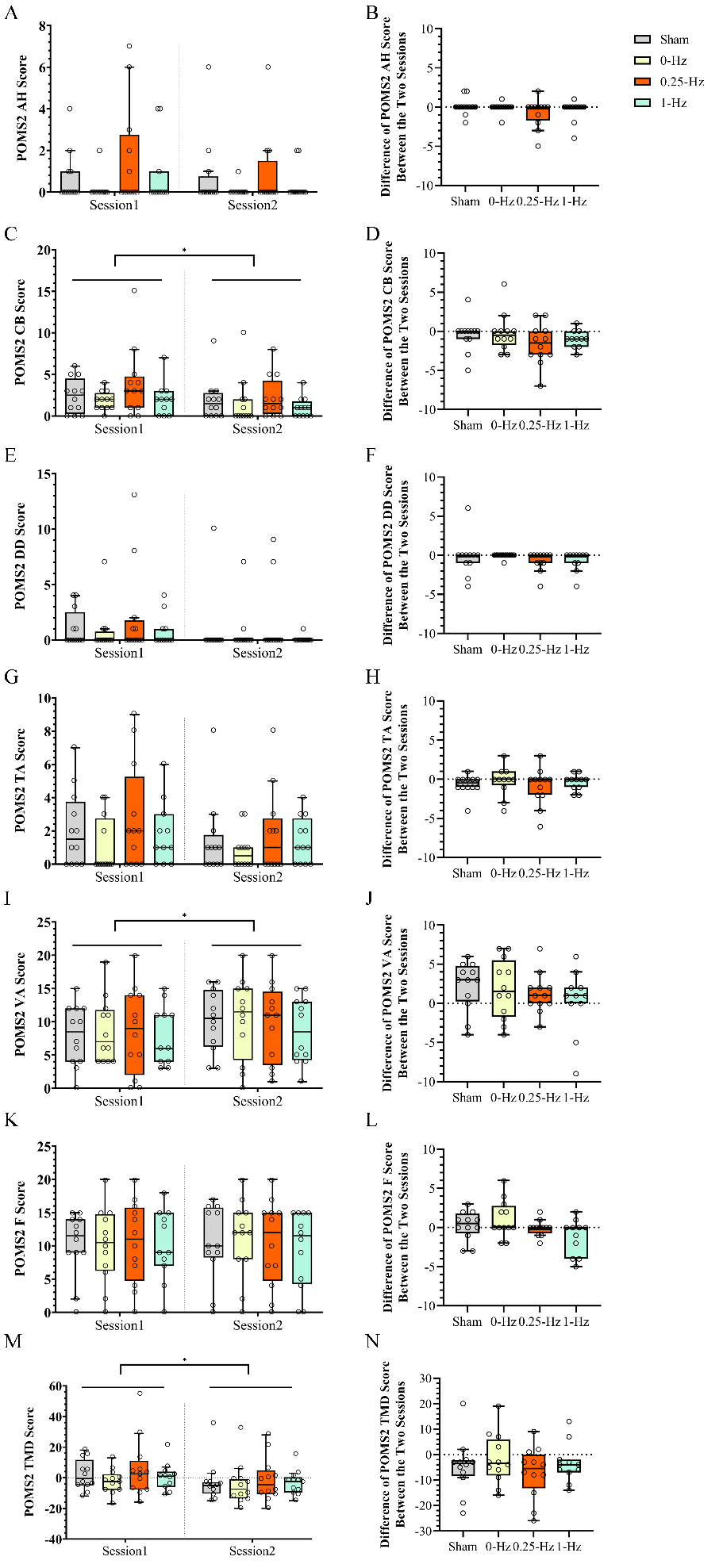


**Figure S2**. Scores of the other scales of the POMS2

Session 1: before-nap sessions and Session 2: after-nap sessions. (A, C, E, G, I, K, and M) Scores in the two task sessions among the four conditions for AH, CB, DD, TA, VA, F, and TMD. (B, D, F, H, J, L, and N) Differences in the two task sessions’ scores between the four conditions for AH, CB, DD, TA, VA, F, and TMD. The box plot shows the distribution of individual data points. The top, bottom, and line in the middle of the box shown in the sequence represent the 75^th^, 25^th^, and 50^th^ percentiles, respectively. The whiskers represent the highest and lowest values that are not outliers or extreme. Circles beyond the whiskers correspond to outliers and extreme values.

POMS2, Profile of Mood States Second Edition-Adult Short; AH, Anger-Hostility; CB, Confusion-Bewilderment; DD, Depression-Dejection; TA, Tension-Anxiety; VA, Vigor-Activity; F, Friendliness; and TMD, Total Mood Disturbance. * p<0.05.


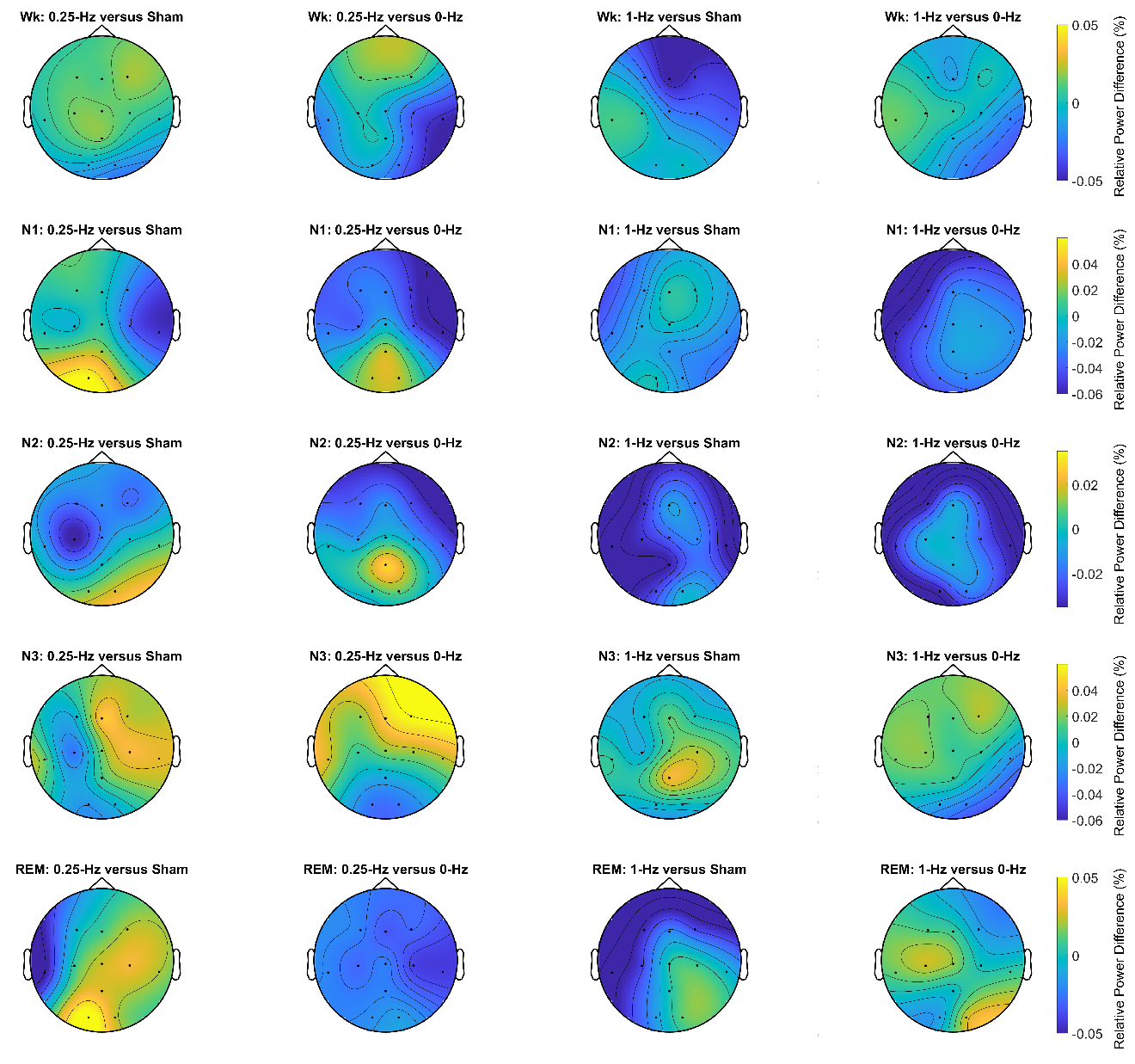


**Figure S3.** Topographic map illustrating the comparisons of relative power at 1 Hz for four pairs: 0.25-Hz BB vs. the sham; 0.25-Hz BB vs. 0-Hz BB; 1-Hz BB vs. the sham; and 1-Hz BB vs. 0-Hz BB conditions, during five sleep stages: Wk, N1, N2, N3, and REM

The rows represent the sleep stages, whereas the columns correspond to the four pairs of comparisons. Dots represent electrodes showing differences in relative 1 Hz power (p>0.05 for black dots, cluster-based permutation test controlling for multiple comparisons). Color intensity reflects the magnitude of differences: yellow signifies a positive difference, whereas blue denotes a negative difference.


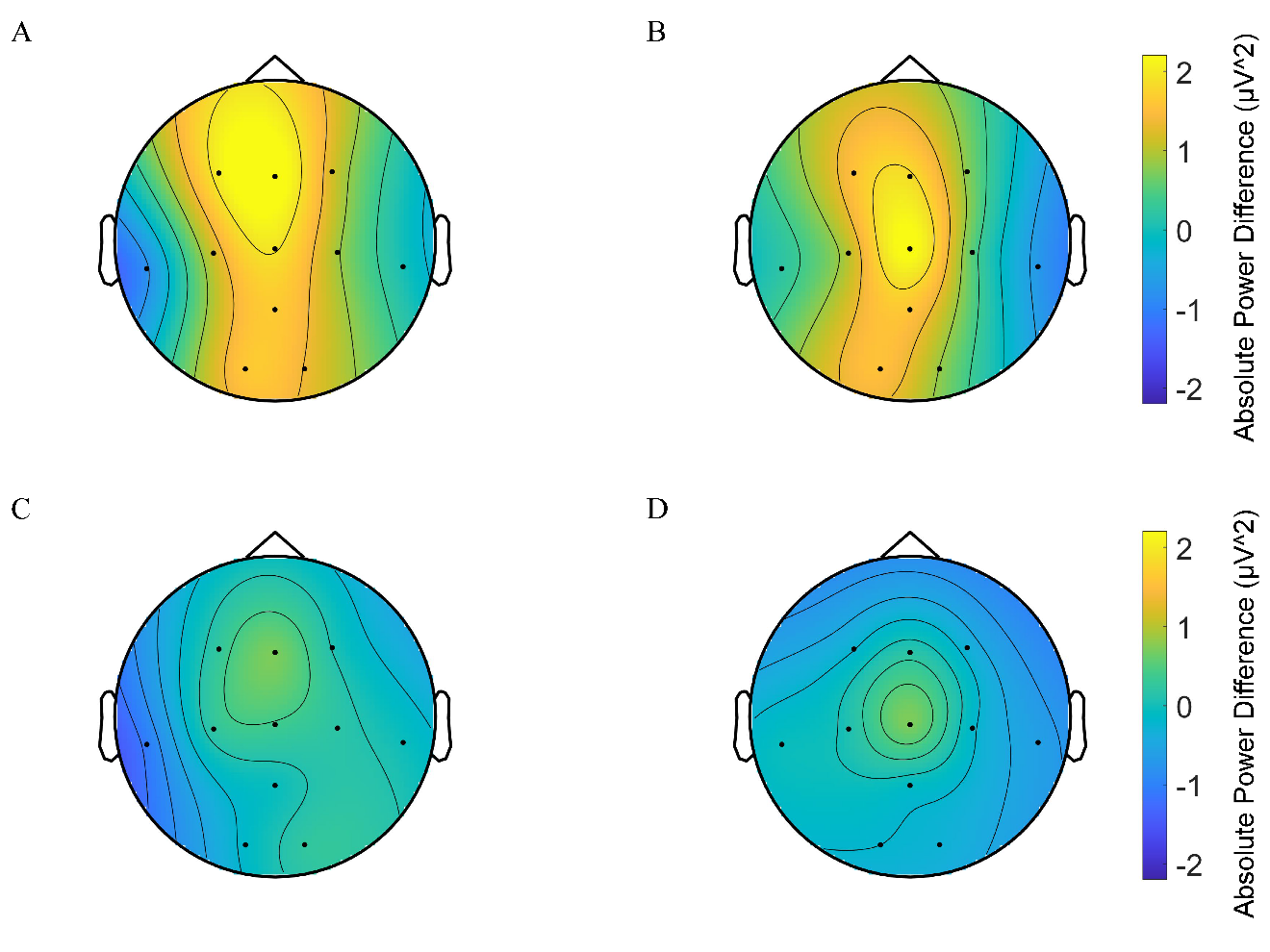


**Figure S4.** Topographic map illustrating the comparisons of absolute delta power for four pairs: (A) 0.25-Hz BB vs. the sham, (B) 0.25-Hz BB vs. 0-Hz BB, (C) 1-Hz BB vs. the sham, and (D) 1-Hz BB vs. 0-Hz BB conditions

Dots represent electrodes showing differences in absolute delta power (p>0.05 for black dots, cluster-based permutation test controlling for multiple comparisons). Color intensity reflects the magnitude of differences: yellow signifies a positive difference, whereas blue denotes a negative difference.


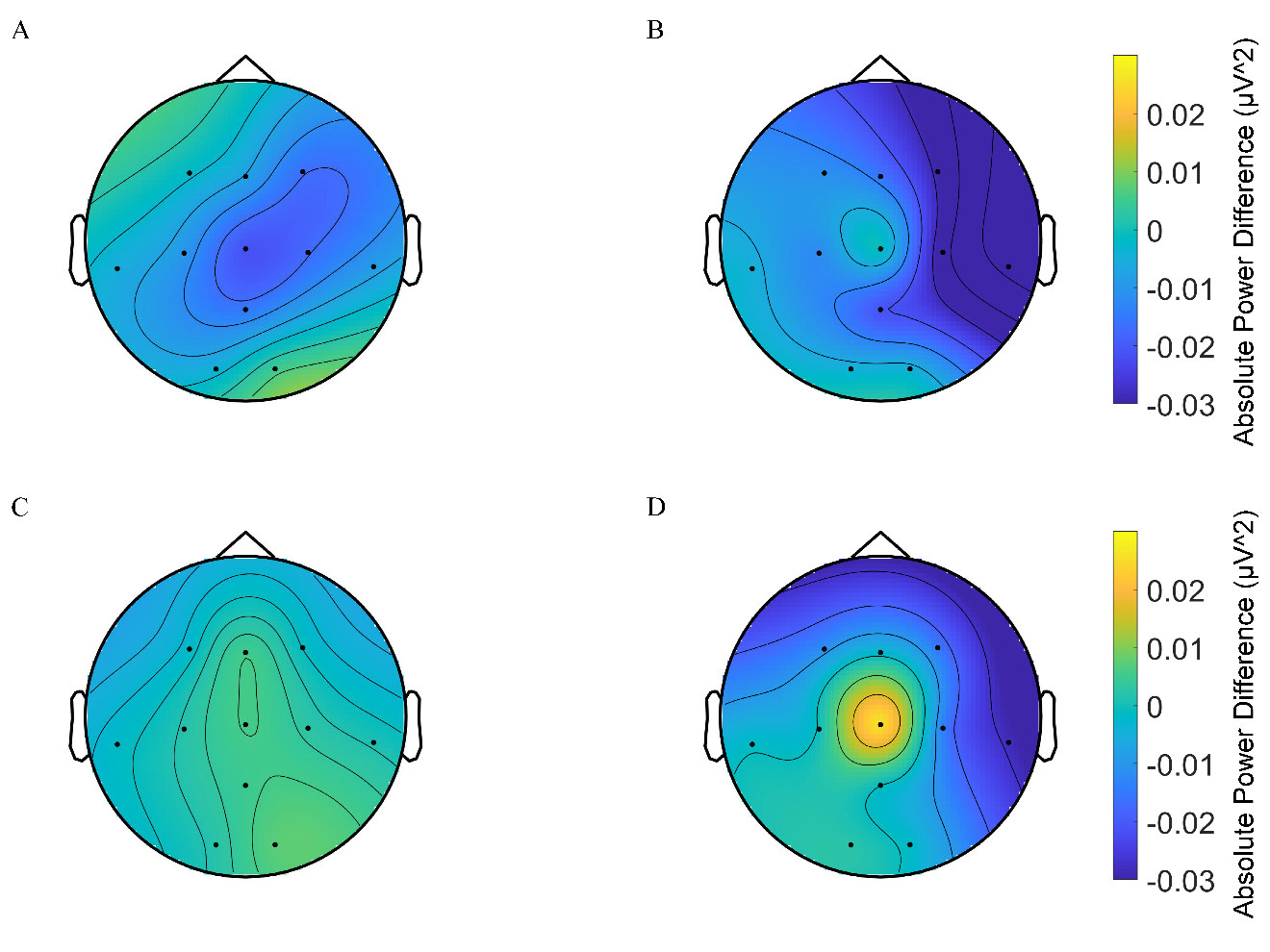


**Figure S5.** Topographic map illustrating the comparisons of absolute sigma power for four pairs: (A) 0.25-Hz BB vs. the sham, (B) 0.25-Hz BB vs. 0-Hz BB, (C) 1-Hz BB vs. the sham, and (D) 1-Hz BB vs. 0-Hz BB conditions

Dots represent electrodes showing differences in absolute sigma power (p>0.05 for black dots, cluster-based permutation test controlling for multiple comparisons). Color intensity reflects the magnitude of differences: yellow signifies a positive difference, whereas blue denotes a negative difference.


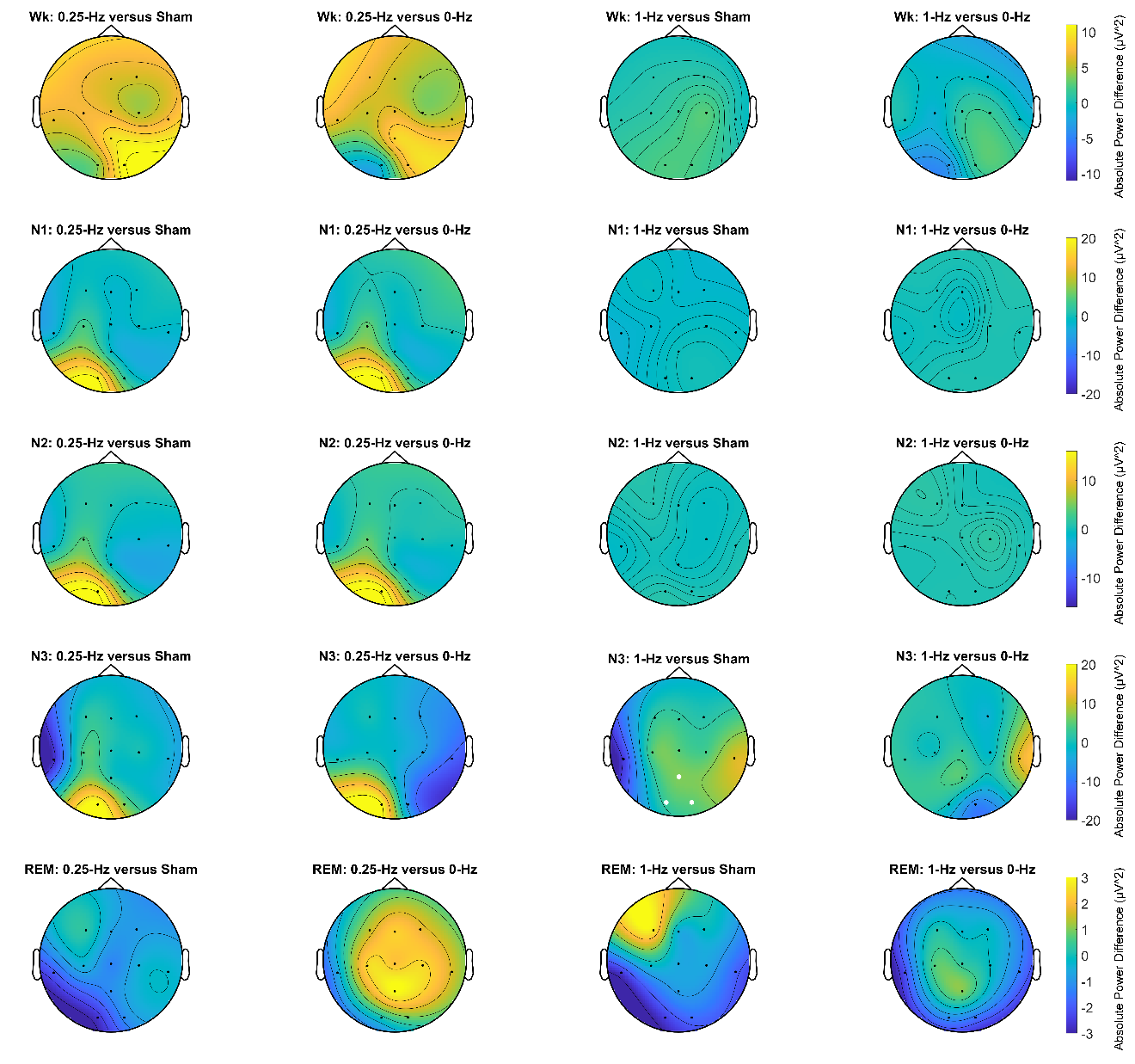


**Figure S6.** Topographic map illustrating the comparisons of absolute power at 0.25 Hz for four pairs: 0.25-Hz BB vs. the sham; 0.25-Hz BB vs. 0-Hz BB; 1-Hz BB vs. the sham; and 1-Hz BB vs. 0-Hz BB conditions, during five sleep stages: Wk, N1, N2, N3, and REM

The rows represent the sleep stages, whereas the columns correspond to the four pairs of comparisons. Dots represent electrodes showing differences in absolute 0.25 Hz power (p>0.05 for black dots, and p<0.05 for white dots, cluster-based permutation test controlling for multiple comparisons). Color intensity reflects the magnitude of differences: yellow signifies a positive difference, whereas blue denotes a negative difference.


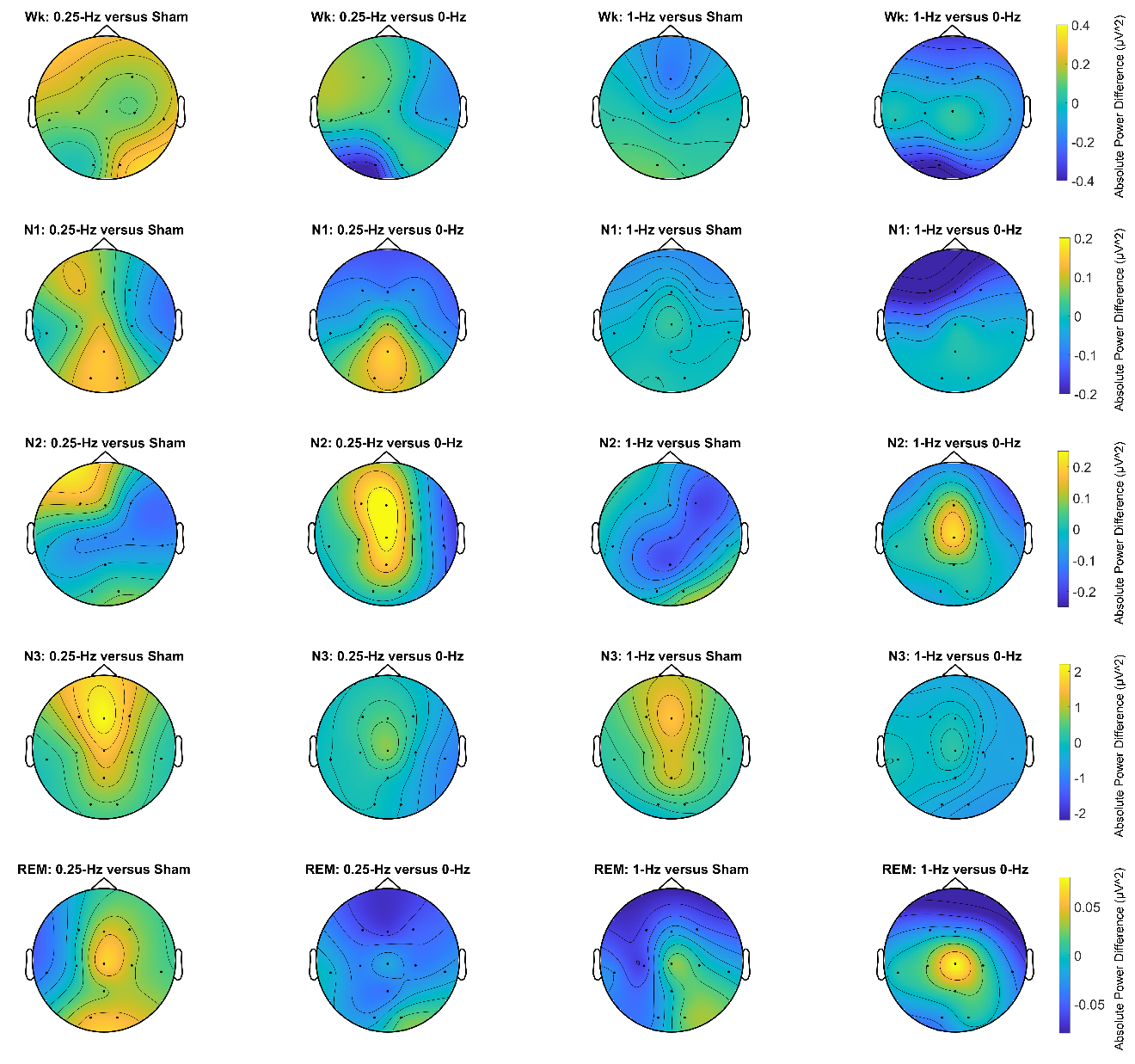


**Figure S7.** Topographic map illustrating the comparisons of absolute power at 1 Hz for four pairs: 0.25-Hz BB vs. the sham; 0.25-Hz BB vs. 0-Hz BB; 1-Hz BB vs. the sham; and 1-Hz BB vs. 0-Hz BB conditions, during five sleep stages: Wk, N1, N2, N3, and REM

The rows represent the sleep stages, whereas the columns correspond to the four pairs of comparisons. Dots represent electrodes showing differences in absolute 1 Hz power (p>0.05 for black dots, cluster-based permutation test controlling for multiple comparisons). Color intensity reflects the magnitude of differences: yellow signifies a positive difference, whereas blue denotes a negative difference.


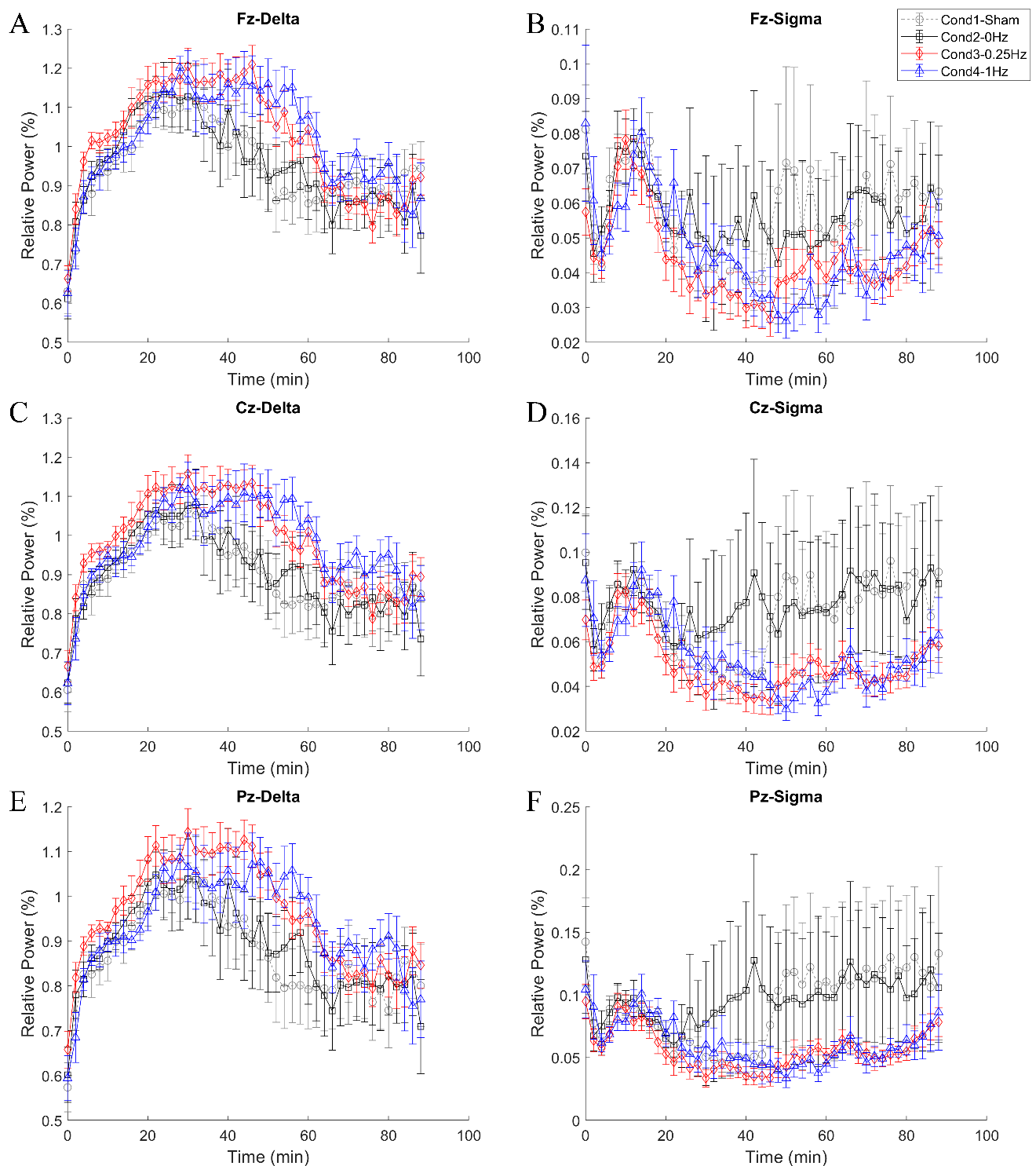


**Figure S8.** Time course of relative delta and sigma power at three representative electrodes Fz (A, B), Cz (C, D), and Pz (E, F) throughout the 90-minute nap period, from lights out to lights on

The rows indicate the time intervals, and the columns represent the relative power values. The line graphs use distinct symbols and colors to represent the group means for the different BB conditions, with error bars indicating ±1 standard error of the mean (SEM).
